# Flow Diversion as a Definitive Treatment for Recurrently Ruptured A1-A2 Anterior Cerebral Artery Aneurysm Following Clipping and Coiling

**DOI:** 10.7759/cureus.57103

**Published:** 2024-03-28

**Authors:** Felipe Ramirez-Velandia, Michael Young, Omar Alwakaa, Kimberly Han, Christopher S Ogilvy

**Affiliations:** 1 Neurological Surgery, Beth Israel Deaconess Medical Center, Harvard Medical School, Boston, USA

**Keywords:** hemodynamics, flow diversion, recurrence, anterior communicating artery aneurysms, anterior cerebral artery

## Abstract

Even after clipping of intracranial aneurysms, patients may experience incomplete occlusion or the future recurrence of their treated aneurysm. This paper presents a distinctive case of a recurrent A1-A2 anterior cerebral artery aneurysm that underwent four interventions over 16 years. The aneurysm was treated with two clippings, subsequent coiling, and flow diversion for definitive treatment. The challenges encountered in managing bifurcation aneurysms are discussed, emphasizing the importance of considering hemodynamic factors, vessel geometry, and recurrence risk factors in treatment decisions. The case highlights the need for closer follow-up of ruptured bifurcation aneurysms due to the higher likelihood of recurrence. The role of flow diverters in reinforcing vessel anatomy and preventing recurrence is also highlighted.

## Introduction

Intracranial aneurysms (IAs) have been reported to affect 2-4% of the general population, with a predilection for females [1,2]. A major concern of IAs growth is the risk for aneurysm rupture, the most common cause of non-traumatic subarachnoid hemorrhage (SAH). While the risk of rupture is influenced by aneurysmal morphology and patient risk factors, annual rupture rates have been reported to be between 0.54% and 1.3% [3]. This variable prevalence and the consequences associated with SAH, including mortality and morbidity up to 50% and 20% respectively, reaffirm the importance of identifying and managing IAs promptly [4].

Even after treatment, patients may experience incomplete occlusion or the future recurrence of their treated aneurysm. When complete occlusion is not achieved patients often require imaging follow-up and retreatment which increases the morbidity and overall quality of life of patients. Previous analyses have shown complete occlusion after surgical clipping in 96.3% of aneurysms, 89.3% after flow diversion (FD), and 78.9% after coiling [3,5]. Moreover, the recurrence rates have been much higher after coiling alone than with surgical clipping or FD [6]. Given the infrequency of recurrence after surgical clipping, it is important to consider the risk factors that may drive aneurysm recurrence including size, morphology, rupture history, location, and smoking history [7]. Location is particularly important as aneurysms at cerebral arterial bifurcation apexes experience higher recurrence [8]. Since any instance of recurrence puts patients at a heightened risk for rupture, gaining a better understanding of proper management for these recurrent bifurcation aneurysms is essential.

In this paper, we illustrate a distinctive case involving an A1-A2 junction anterior cerebral artery aneurysm that underwent a series of four treatments over a span of 16 years. While the literature extensively documents cases of pipeline embolization as a salvage measure after coiling and clipping, the uniqueness of our case lies in the necessity for multiple interventions to address the morphologic changes and recurring nature of the aneurysm under consideration.

## Case presentation

**Figure 1:**
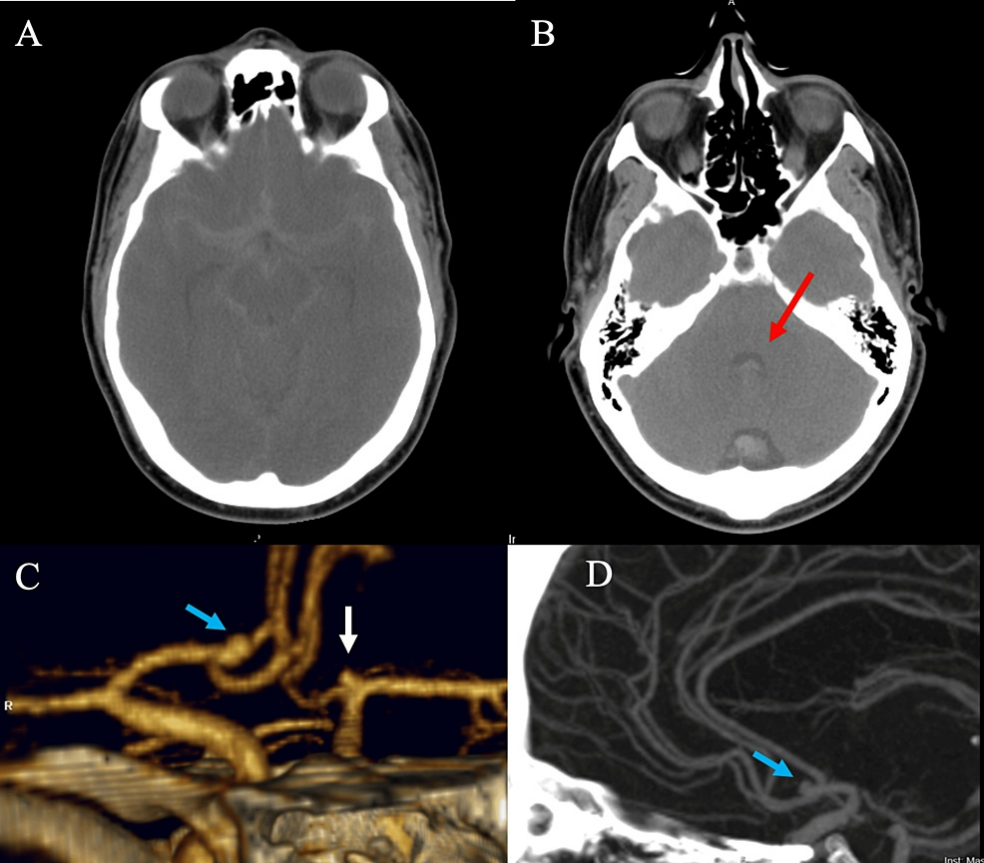
Computed tomographic images at presentation There was evidence of diffuse subarachnoid hemorrhage with extension to the Sylvian fissures, frontal sulci, cerebral hemispheres bilaterally (A), and intraventricular hemorrhage at the fourth ventricle (red arrow; B). CT angiography demonstrated a 3 mm right A1-A2 junction aneurysm, directed medially, anteriorly, and superiorly (blue arrow) and a 3 mm left bifurcation aneurysm (white arrow; C, D).

**Figure 2:**
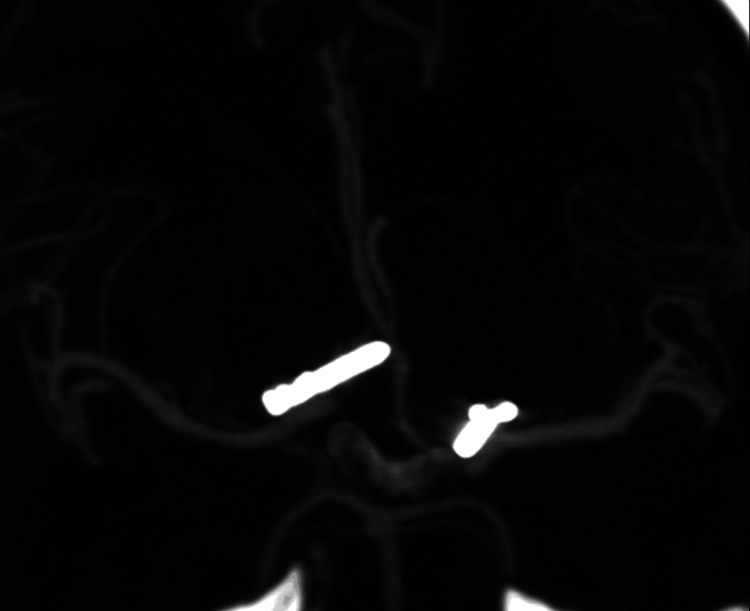
Computed tomographic angiography five days after initial clipping There was evidence of complete obliteration of both clipped aneurysms.

A 51-year-old man presented in 2007 to the emergency department because of worsening headaches after a syncopal episode. CT head at the time of presentation demonstrated diffuse SAH with intraventricular extension (Figures 1A-1B). Further evaluation through angiography identified a right A1-A2 junction aneurysm and a 3 mm left internal carotid artery (ICA) terminus aneurysm (Figures 1C-1D). The patient underwent clipping of the right A1-A2 junction aneurysm due to the concern that this was the ruptured aneurysm based on the pattern of SAH. Two days later, the patient underwent clipping of the left ICA terminus aneurysm. Follow-up CT angiography (CTA) demonstrated no residual filling of the right A1-A2 junction aneurysm or the left ICA terminus aneurysm (Figure 2). The patient was discharged home two weeks later with headaches and mild cognitive issues (modified Rankin Scale (mRS)=1).

**Figure 3:**
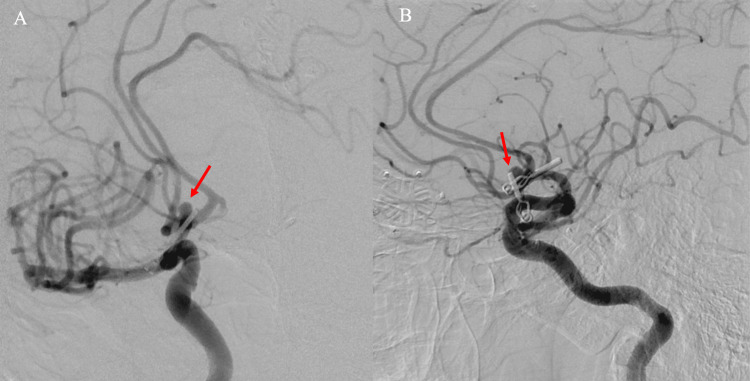
Digital subtraction angiography performed five years after initial clipping On the anteroposterior (A) and lateral views (B), there was evidence of recurrence of the A1-A2 aneurysm arising posterior to the site of clipping and measuring 4x5x4 mm (red arrow).

**Figure 4:**
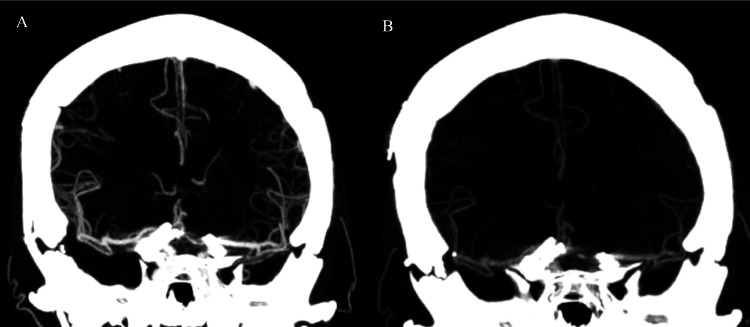
Computed tomographic angiographies after the second microsurgical treatment In the angiographies performed at one (A) and three years (B) after the second clipping, there was no evidence of residual filling in the recurrent A1-A2 aneurysm.

**Figure 5:**
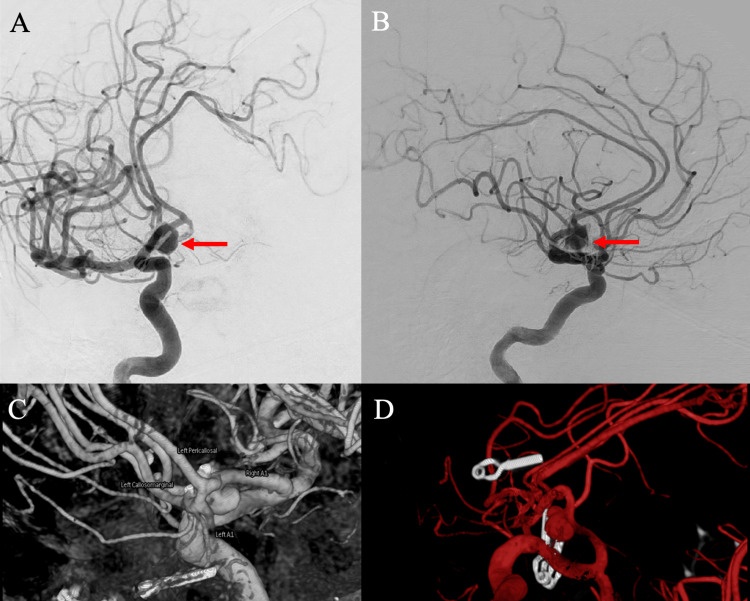
Digital subtraction angiography performed 15 years after initial A1-A2 clipping and 10 years after the second clipping for recurrence In the anteroposterior (A) and lateral (B) runs, there was evidence of a new recurrence of the anterior communicating artery (ACOM) aneurysm, displaying a small fusiform bulge between the two clips, and inferior projection into the prior site of clipping and measuring 6.7 mm in maximum diameter (red arrows). Further 3D reconstructions were performed to characterize the aneurysm and evaluate its relationship with nearby vessels (C, D).

**Figure 6:**
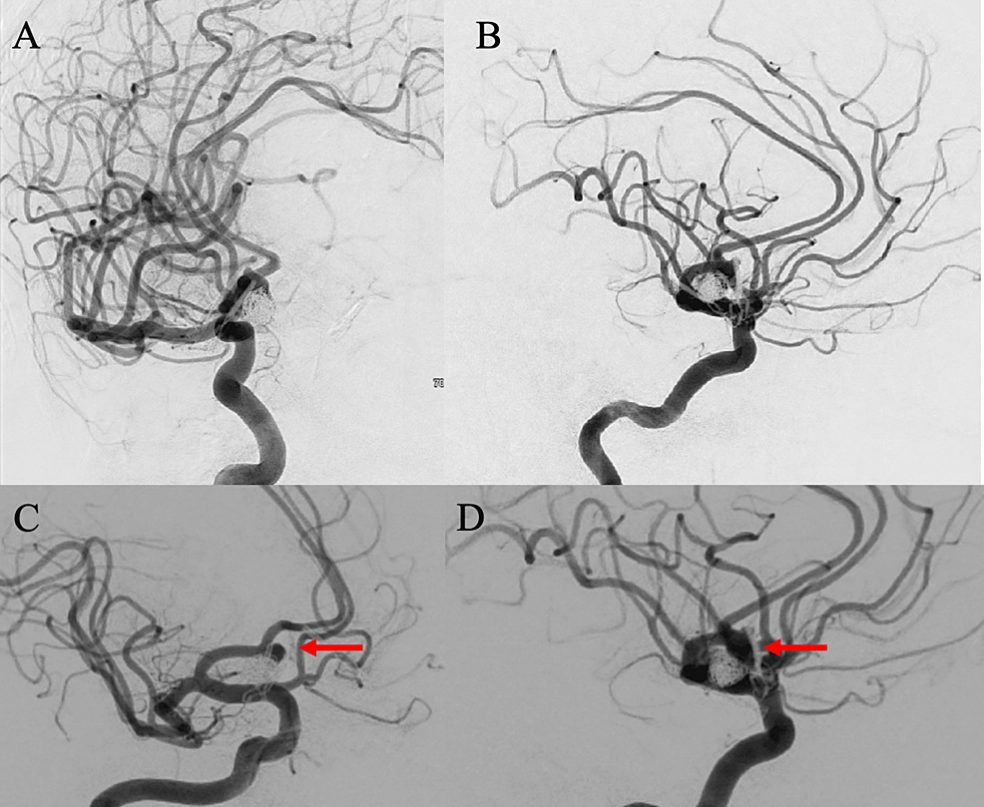
Digital subtraction angiography runs after coiling embolization of the new recurrence Anteroposterior (A) and lateral (B) injections demonstrated successful Raymond Roy 2 occlusion of the recurrent anterior communicating artery (ACOM) aneurysm that measured 8.8 mm in maximal diameter on the day of the intervention. Unfortunately, one year after this procedure there was new angiographic evidence of residual filling of the dome of the aneurysm evidenced in the oblique (C) and lateral (D) angiographic runs (red arrows).

In 2012, the patient underwent follow-up digital subtraction angiography (DSA) which demonstrated a new recurrence measuring 4mm x 5mm x 4mm projecting posteriorly to the previous clip (Figure 3). At this time, he underwent clipping of the recurrent, previously ruptured right A1-A2 aneurysm. Follow-up CTA done in 2013 and 2015 demonstrated no residual right A1-A2 aneurysm or left ICA terminus aneurysm (Figure 4). However, in the follow-up DSA in 2022, there was evidence of a new outpouching at the A1-anterior communicating artery (ACOM) complex; this time the aneurysm displayed a different morphology and was facing inferiorly and posteriorly (Figure 5). After a multidisciplinary discussion, a consensus was reached to proceed with the coiling of this recurring aneurysm. The patient underwent primary coiling and at the end of the procedure, there was minimal filling at the neck of the aneurysm (Raymond-Roy grade 2 occlusion; Figure 6).

**Figure 7:**
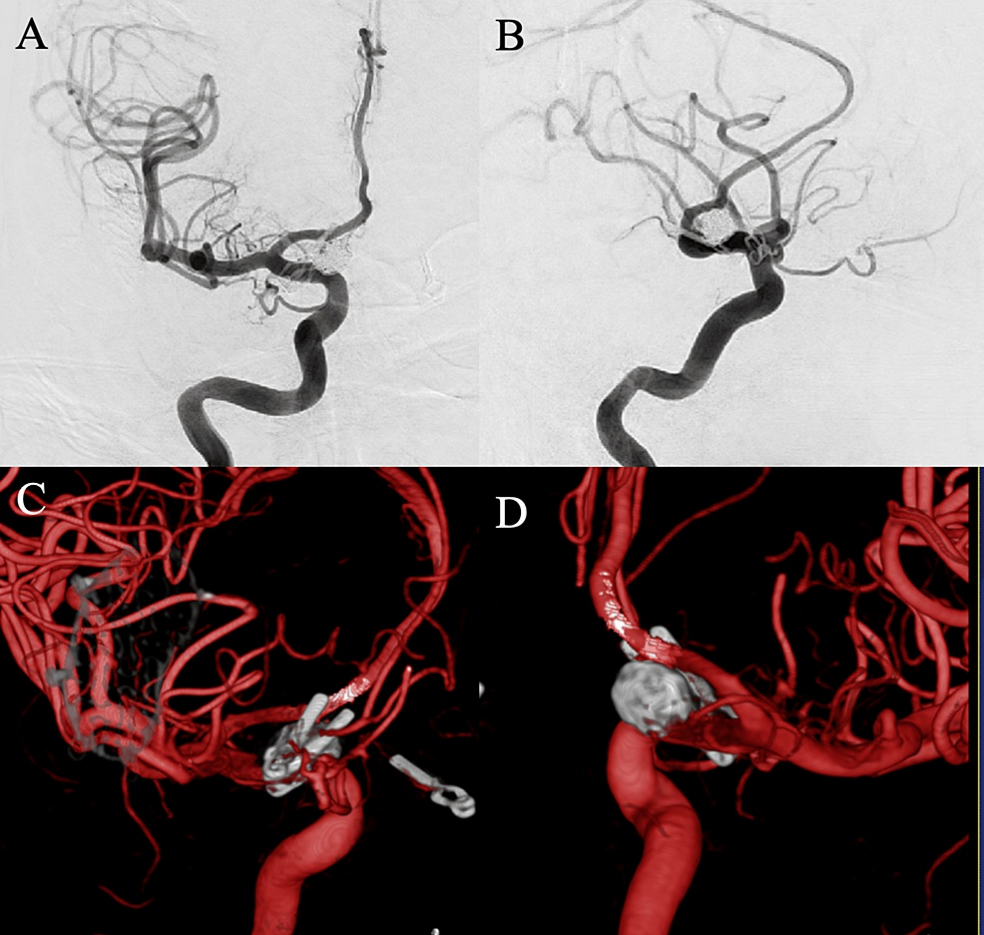
Digital subtraction angiography performed one year after treatment with flow diversion Anteroposterior (A), lateral (B), and 3D reconstruction images (C, D) demonstrated complete occlusion of the recurrent aneurysm one year after treatment with a pipeline embolization device.

In 2023, there was new angiographic evidence of growth of the residual recurrent aneurysm, measuring 6 mm in its maximal diameter. Again, after a multidisciplinary discussion, the decision was made to attempt FD from the right A1 segment of the anterior cerebral artery to the right A2 segment of the anterior cerebral artery. The patient underwent successful FD with a pipeline embolization device (PED) of this recurrence, and on a one-year follow-up DSA, there was complete occlusion of the aneurysm (Figure 7).

## Discussion

Bifurcation aneurysms, representing under two-thirds of all IAs, pose a formidable challenge in cerebrovascular treatment owing to their anatomy, hemodynamic characteristics, and their tendency to recur. High wall shear stress (WSS) plays a significant role in the development and growth of bifurcation aneurysms. WSS is described as stress induced by turbulence in the flow. At sites of bifurcation, the flow of the branching vessels is perpendicular to the apex wall, causing a change in the laminar flow [9]. This stress could act on the shape and structure of endothelial cells and cause a loss of mechanical resistance of the vessel wall [8]. This effect may lead to the growth of aneurysms and could also impact hemodynamic parameters in both the local and neighboring arteries [10].

Hemodynamic changes can also influence the post-treatment pathogenesis of bifurcation aneurysms [8]. In a computational flow dynamics (CFD) simulation by Ortega et al., it was demonstrated that after endovascular treatment of basilar apex aneurysms, there was a significant rise in WSS in the parent vessel after achieving complete aneurysm occlusion [11]. This effect could lead to permanent remodeling in the arterial wall in the adjacent area of the treated aneurysm neck. As a result, a recurrent aneurysm could develop near the treated aneurysm [10,11]. Similarly, clipping can induce hemodynamic changes in the adjacent vessels creating areas of adjacent WSS within the parent vessel [10].

The application of surgical clipping as a therapeutic intervention for IAs has been a longstanding practice, tracing its origins to the first case performed by Dandy in 1937 [12]. Clipping offers the unique advantage of securing the neck of the aneurysm with a pre-established pressure, thereby achieving long-term occlusion. Completely clipped IAs have a considerably low risk of recurrence. However, in many cases, this is not possible due to the complexities of the aneurysms. Moreover, in literature, it has been suggested that clipping can exhibit an annual recurrence rate of 0.26-0.53% and can even trigger de novo aneurysms adjacent to the site of occlusion [13,14]. Although this is true, some aneurysms can recur quicker secondary to clip slippage. Factors implicated in these recurrences include incomplete neck coverage, larger aneurysm sizes, and the presence of multiple aneurysms [15,16]. Other authors have pointed out that A1-ACOM aneurysms pointing anteriorly, and smoking are additional factors predicting recurrence after surgical treatment [17].

In the ACA bifurcation specifically, studies have shown that enlarged ACA bifurcation angles can lead to abnormally enhanced hemodynamic stresses [18]. In a CFD evaluation including 115 aneurysms located in the ACA bifurcation, it was identified that the geometry and the bifurcation angle were strong determinants of WSS [18]. Interestingly, in this analysis, ACA bifurcation aneurysms demonstrated a smaller lateral bifurcation angle towards the smaller bifurcation branch. This validates the findings from other cohorts and the presented case, in which the aneurysm tends to be lateralized to the smaller branch of the bifurcation [19]. The authors concluded that stenting at sites of bifurcations can significantly increase the lateral angle and decrease the bifurcation angle, which will displace and attenuate the flow-impinging zone, ultimately decreasing the WSS and total pressure at the bifurcation apex. In fact, stent-assisted technologies have been shown to reduce the hemodynamic stress of bifurcation aneurysms, through angle remodeling [19,20].

Flow diverter stents (FDS) confer a distinctive advantage of seemingly incorporating into the vessel wall after endothelialization of the stent struts occurs [21]. Therefore, in addition to inducing changes in flow impedance and promoting aneurysm sac thrombosis, the device plays a role in reestablishing vessel architecture, minimizing the risk of recurrence after complete aneurysm obliteration. Incomplete aneurysm occlusion after FD, a phenomenon occurring after 16.4% to 32% of procedures, has been related to age, comorbidities, smoking history, aneurysm size, morphology, the location of branches arising from the aneurysm neck or dome, and device malposition [22,23]. Retreatment rates after FD are much lower, and reported after 5.7% of procedures [24].

FD has been well recognized as a salvage therapy used when other alternatives prove to be ineffective. Adeeb described seven aneurysms with recurrence 13 years after clipping and treated successfully with a PED [25]. To note, none of these aneurysms were located in the ACA bifurcation. More recently, in a case series by Heiferman et al., including a total of 25 aneurysms treated with FD after ineffective stent-assisted coiling, 76% of the aneurysms showed improvement in the Raymond Roy classification [26]. Moreover, according to a retrospective analysis performed at our institution including patients treated with PEDs in a span of 10 years and achieving 84% complete or near occlusion, it was identified that 15.7% of aneurysms treated with FD had prior endovascular treatment or clipping [27].

In the literature we reviewed, there are only a few cases of recurrence after clipping of ACA-ACOM complex aneurysms [28,29]. Our presented case contributes to the literature by highlighting the uncommon recurrence after successful clipping and elucidating the challenges associated with hemodynamic factors intrinsic to bifurcation aneurysms. Bifurcation aneurysms may require closer follow-up due to their higher likelihood of recurrence over time. However, additional cost-effectiveness analyses are needed to identify the optimal timing for this follow-up.

## Conclusions

In conclusion, this case of an A1-A2-ACOM aneurysm, which recurred despite traditional clipping and coiling methods, highlights the challenges encountered in managing bifurcation aneurysms. Factors contributing to recurrence in our patient included the location and weakened vessel wall from a diseased vessel. The widespread use of FD has offered hope in addressing the complexities of certain IAs such as the one described in the following case. FD provides the unique advantage of reinforcing vessel anatomy and preventing recurrence.
